# The 3D hype: Evaluating the potential of real 3D visualization in geo-related applications

**DOI:** 10.1371/journal.pone.0233353

**Published:** 2020-05-21

**Authors:** Vojtěch Juřík, Lukáš Herman, Dajana Snopková, Adrianne John Galang, Zdeněk Stachoň, Jiří Chmelík, Petr Kubíček, Čeněk Šašinka

**Affiliations:** 1 Department of Psychology, Faculty of Arts, Masaryk University, Brno, Czech Republic; 2 Department of Geography, Faculty of Science, Masaryk University, Brno, Czech Republic; 3 Department of Computer Graphics and Design, Faculty of Informatics, Masaryk University, Brno, Czech Republic; 4 Department of Information and Library Studies, Faculty of Arts, Masaryk University, Brno, Czech Republic; University of Florida, UNITED STATES

## Abstract

The use of 3D visualization technologies has increased rapidly in many applied fields, including geovisualization, and has been researched from many different perspectives. However, the findings for the benefits of 3D visualization, especially in stereoscopic 3D forms, remain inconclusive and disputed. Stereoscopic “real” 3D visualization was proposed as encouraging the visual perception of shapes and volume of displayed content yet criticised as problematic and limited in a number of ways, particularly in visual discomfort and increased response time in tasks. In order to assess the potential of real 3D visualization for geo-applications, 91 participants were engaged in this study to work with digital terrain models in different 3D settings. The researchers examined the effectivity of stereoscopic real 3D visualization compared to monoscopic 3D (or pseudo 3D) visualization under static and interactive conditions and applied three tasks with experimental stimuli representing different geo-related phenomena, i.e. objects in the terrain, flat areas marked in the terrain and terrain elevation profiles. The authors explored the significant effects of real 3D visualization and interactivity factors in terms of response time and correctness. Researchers observed that the option to interact (*t* = -10.849, *p* < 0.001) with a virtual terrain and its depiction with real 3D visualization (*t* = 4.64, *p* < 0.001) extended the participants’ response times. Counterintuitively, the data demonstrated that the static condition increased response correctness (*z* = 5.38, *p* < 0.001). Regarding detailed analysis of data, an interactivity factor was proposed as a potential substitute for real 3D visualization in 3D geographical tasks.

## Introduction

3D visualizations are being increasingly used in a number of applied areas for data visualization. Since these visualizations allow three-dimensional perception of graphical content, they are considered a promising tool for a range of applications in geo-sciences, for example, teaching geography and cartography [1–5], urban planning [6–8], crisis management [9–12], precision agriculture [13], visibility analysis [14], virtual tourism [15], navigation in built-up areas [16, 17], indoor navigation [18, 19] and others. Different forms of 3D visualization may encourage different types of human behavioural and cognitive responses, i.e. they can affect human sensorimotor and interaction strategies, cognitive processing, and ultimately, human performance. The aim of this study was to evaluate important factors contributing a role in 3D geovisualizations, specifically 3D factors and interactivity factors in the context of different geo-related tasks. In this study, 3D geovisualization is understood as a three-dimensional visual representation of the real world, its parts, or as a representation of the spatially referenced data [20]. The 3D geovisualization may be of a dynamic nature, so it allows for changes of depiction based on the user-computer interaction. Regarding this, 3D geovisualization allows the user to focus on its specific parts or aspects from various positions, perspectives, and other functionalities (see [21]). In this study, authors explored participants response times and accuracy of answers in different forms of geovisualization, specifically focusing on the level of interactivity and 3D visualization type, as stated in detail below in the research hypotheses.

### 3D visualization types

The types of 3D visualizations vary in the principles that visualizations are built on and the technologies used to display them. The most typical types of 3D visualizations are pseudo 3D visualizations (also known as weak or 2.5D visualizations) and the less common real (or strong) 3D visualizations [22, 23]. Pseudo 3D visualizations depict a scene which is displayed perspective-monoscopically on flat media, such as computer screens or widescreen projection. These scenes are composed solely with the use of monocular depth cues [23]. Real 3D visualizations engage both monocular and binocular depth cues (especially binocular disparity cues) in order to achieve stereoscopic vision. Stereoscopy is a technique using stereopsis [24] to separate the visual signals individually perceived by each eye and present them through a peripheral device, for example, 3D shutter glasses [25]. The slight differences in perspective in image between the left and the right eye are registered and combined to create a 3D representation of the observed scene. Real 3D vision was developed to enhance perception of spatial attributes in an observed scene, i.e. the distances and relative positions of objects in a visual field [26, 27], and to increase depth perception in a scene [28]. Real 3D technology provides a different number of visual cues than pseudo 3D, and therefore, it is expected that the displayed content is processed differently with respect to the visualization type. Hence, real 3D technology has also been proposed as a more effective tool for virtual geovisualization since it provides additional spatial information [1, 29, 30], even though no clear standards for the production and use of real 3D visualizations in geo-sciences are available [22, 23, 31]. Despite the added visual cues in real 3D visualization, the effectiveness and efficiency [32, 33] of stereoscopic 3D visualization in applied issues remains disputed, especially regarding increased response time to solve tasks in a real 3D environment, increased cognitive load and user distraction, neglect of important objects in the scene, or significant visual discomfort while wearing peripheral devices, such as 3D glasses or helmets [23, 28, 34–36]. Several empirical studies support the effectiveness of real 3D visualization [37–40], although their practical use, especially in geo-related issues, remains controversial [30, 41].

### Interactivity factor

**Fig 1:**
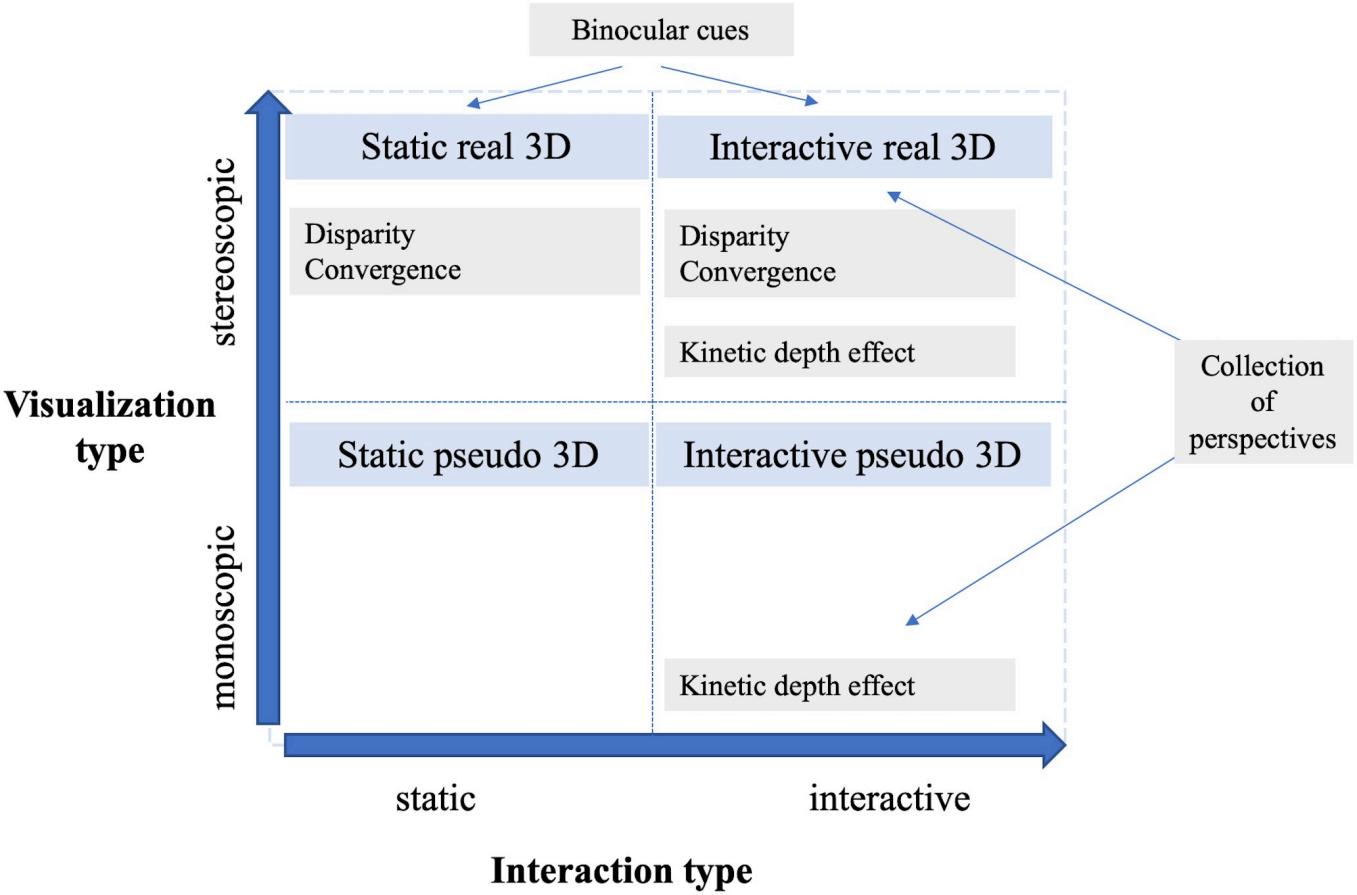
Scheme of the additional depth cues present in various settings.

The shift from static maps to interactive versions is a natural step, as technological progress permits and drives it. Interactivity in current geospatial data visualizations is starting to become available for users to navigate or otherwise work with data to obtain required information and acquire optimal situation awareness [42, 43]. In the present paper, the researchers focused on navigational (or viewpoint) interactivity, which was discussed in previous studies [43–46], and which forms the core functionality of applications such as Google Earth and other virtual globes. Navigational interactivity in 3D space usually includes functions such as rotation, panning and zooming for spatial information acquisition further promoting e.g. spatial orientation [46]. Previous studies [19, 45, 47–49] suggested a difference between static and interactive scenes in perception and cognitive processes, and respectively emphasized the importance of the specific task type [45]. Interaction with a geovisualization helps complete the spatial information about the scene as the scene is moved and presented from various points of view. Interaction provides a collection of perspectives, and hence the mental model of the scene can be more rapidly processed by the observer, as previously discussed by [36, 50, 51]. Other authors have suggested that interaction only improves task solving in a limited manner since users need not be able to use it properly [52]. Regarding this, the present study questions the role of 3D geovisualizations in the context of interactive and static tasks and suggests that real 3D visualization can be possibly substituted with the option to control visualized content. The summary of the available depth cues present in various settings is shown in Fig 1.

### Geographical task factor

A number of studies explored simple 3D geovisualizations [36, 45, 53, 54]. Other studies explored complex stimuli similar to real-world settings [4, 19, 55]. Previous findings suggested that the role of 3D technology in geovisualizations is questionable, especially regarding the specific features of the user interface (interactivity factor, type of task, etc.). Research in geo-applications emphasizes the role of the specific task since there is very limited option to control the equivalence of stimuli—a real map is a unique combination of spatial aspects in which it is difficult to find equivalent stimuli. For this reason, the authors developed an experiment that uses three types of tasks as experimental stimuli (i.e. point, surface and elevation profile structures in the virtual terrain), which represent different geo-related phenomena, such as buildings, water bodies and roads. Participants were asked to determine the elevation of objects (cubes), water bodies and the presented terrain profiles in the virtual terrain. These three task types possessed different spatial complexity and represented different spatial structures. As spatially simple and complex stimuli, the cubes and terrain profiles, respectively, had already been tested in previous studies [36, 49] and were complemented in the present study with semi-complex “surface” stimuli, i.e. water bodies.

### Research aims

The present study explored the effectivity of real 3D geovisualization in static and interactive tasks. Effective form does not refer to the simplest/easiest form but to one that offers users sufficient information necessary to make a proper choice and prevent mistakes while also facilitating quick response, i.e. it balances effectiveness (score) and efficiency (speed). Quesenbery [56] suggested three general aims that every user interface should follow: (a) effectiveness in achieving determined objectives, usually completing the task (i.e. correctness), (b) efficiency in control or use (i.e. speed and ease), and (c) the operator’s satisfaction in working with such a device. Regarding these points, the suitability of a specific user interface setting can be operationalized and tested. The present study examined the first two above-mentioned points, i.e. goal achievement and speed, as these can be operationalized into objective performance scores and response times in the suggested geo-related tasks. As mentioned in more detail above, previous studies have suggested that real 3D visualization is potentially troublesome and may cause visual discomfort, prolong solution time or promote neglect of important objects in a scene. The aim of this study was to assess the effect of 3D visualization on goal achievement and performance speed under different 3D conditions with different tasks. One of the main questions was the role of real 3D technology in the context of interactive geographic tasks, in which navigational interactivity [43] was suggested as being able to potentially compensate for missing binocular cues (as discussed in [36]). Regarding this, the authors of the present study observed user performance in real 3D, and respectively, pseudo 3D visualizations, both in static (i.e. non-interactive) and interactive environments. The present study is specific in several ways. Compared to the previous research of [28, 57], the authors inspected the effect of interactive 3D visualizations in virtual models of real, existing geographical areas. Previous studies also engaged a limited number of participants, generally only up to 20 people [58–60], thereby limiting general conclusions. Furthermore, the present study emphasized the process of interaction with terrains generated by the participants themselves and avoided the use of automated computer-generated movement.

The research hypotheses were:

- Tasks in a real 3D environment require a longer time to be solved,
- Tasks in an interactive environment require a longer time to be solved,
- Tasks in a real 3D environment result in more correct answers,
- Tasks in an interactive environment result in more correct answers.

## Methods

### Participants

The previous study [36] suggested that the 3D visualization type had a significant effect in identifying an object’s elevation in a static (non-interactive) 3D geovisualization (*Cohen’s d* = 1.06). The authors of the present study considered the experiment’s design (e.g. computer screens instead 3D wide-screen projection) and other observations concerning similar issues [49]. Only a relatively mild effect was expected. Regarding this, 91 participants were engaged in the experiment. Since the study examined the topic of perception, volunteers, specifically humanities students with no or minimal previous training in geo-visualization from Masaryk University (17 males, 75 females), aged 19 to 53 years (*m* = 23.46 years; *med* = 23 years; *sd* = 4.9), were involved. Course-takers of Masaryk University’s Experimental Humanities course, which is held annually at the Faculty of Arts, were invited via email. By participating in the experiment, they were rewarded with 16 points towards the above-mentioned course. Before the experiment, all participants were questioned about visual impairments and any other possible medical limitations and informed that they could remove themselves from the experiment at any time. The study was approved by the Masaryk University Ethics committee for the research, identification number of the project: EKV-2016-059. Participants in the study gave their consent by means of a written consent form.

### Procedure and materials

The entire experiment was fully computerized. The participants used conventional desktop PCs with 27-inch 3D monitors and wore active shutter 3D glasses (NVIDIA 3D Vision^®^ 2 Wireless Glasses, 60 Hz on each eye). Regular optical mouses were used for UI control. The participants were introduced to the experiment and instructed on the control devices, interface and purpose of the experiment. After the introduction, a questionnaire presented on the PC asked for their demographic data and about any visual impairments. After this, participants were instructed how to proceed through the testing and that they should attempt to be fast as well as accurate since both their speed and correctness would be measured. The testing application was created at the Faculty of Informatics (Masaryk University, Brno) specifically for the purpose of the study. The participants were instructed to follow the information on the screen, for example, when to wear or remove the 3D glasses with respect to the specific test section. After instructions, the participants were pseudo-randomly assigned into the specific experimental groups (based on the task type category). The participants then commenced a training session to learn how to navigate/control the interactive geovisualizations and how to put on/take off the 3D glasses. After the training, the participants completed 24 assigned tasks in the experiment. At the end of the experiment, participants were debriefed and rewarded with sweets.

### Research design

The study employed a within-between multi-factorial design. The first factor was *3D visualization type* (two levels: pseudo 3D visualization and real 3D visualization), the second factor was *interactivity type* (two levels: interactive type and static, or non-interactive, type) and the third factor was *task type* (three levels: cubes representing objects, e.g. buildings, surfaces representing water bodies, and terrain profiles representing, e.g. roads). This legend (buildings, water bodies, roads) was described to the participants at the beginning of the experiment to better illustrate the purpose of the experiment and tasks. Taking into account the length of the experiment and potential fatigue in the participants, the research sample was pseudo-randomly divided into three subsamples (regarding the ratio of males and females) with respect to the specific task type (cubes, water bodies, terrain profiles). This meant that each subsample dealt with only one type of task (24 trials). Each task measured (i) response time (in seconds) and (ii) correct elevation estimation (participants were asked to select one of three possible options and scored 1 point for a correct answer and 0 for an incorrect answer). Time, correctness and the number of the mouse clicks participants made was recorded automatically with the software developed for testing.

**Fig 2:**
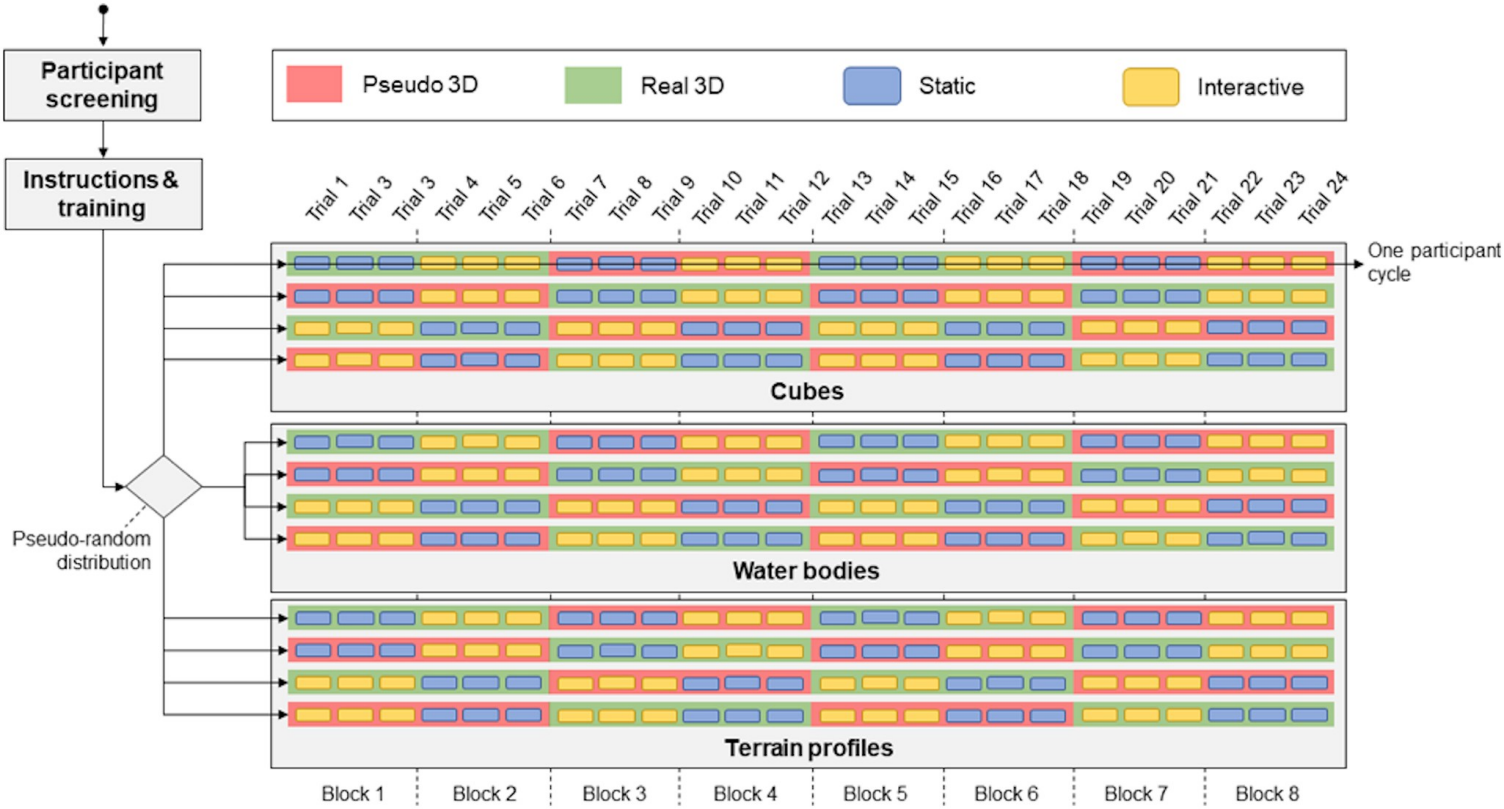
Diagram of the procedure of the experiment.

The research design was balanced, each participant underwent 24 tasks divided into 8 blocks. Each block consisted of 3 trials of the same type (e.g. interactive real 3D). In an effort to reduce the primacy effect, the participants commenced the test’s static real 3D, static pseudo 3D, interactive real 3D and interactive pseudo 3D condition alternately (Fig 2).

### Description of the tasks

#### Comparing the positions of cubes in the terrain

**Fig 3:**
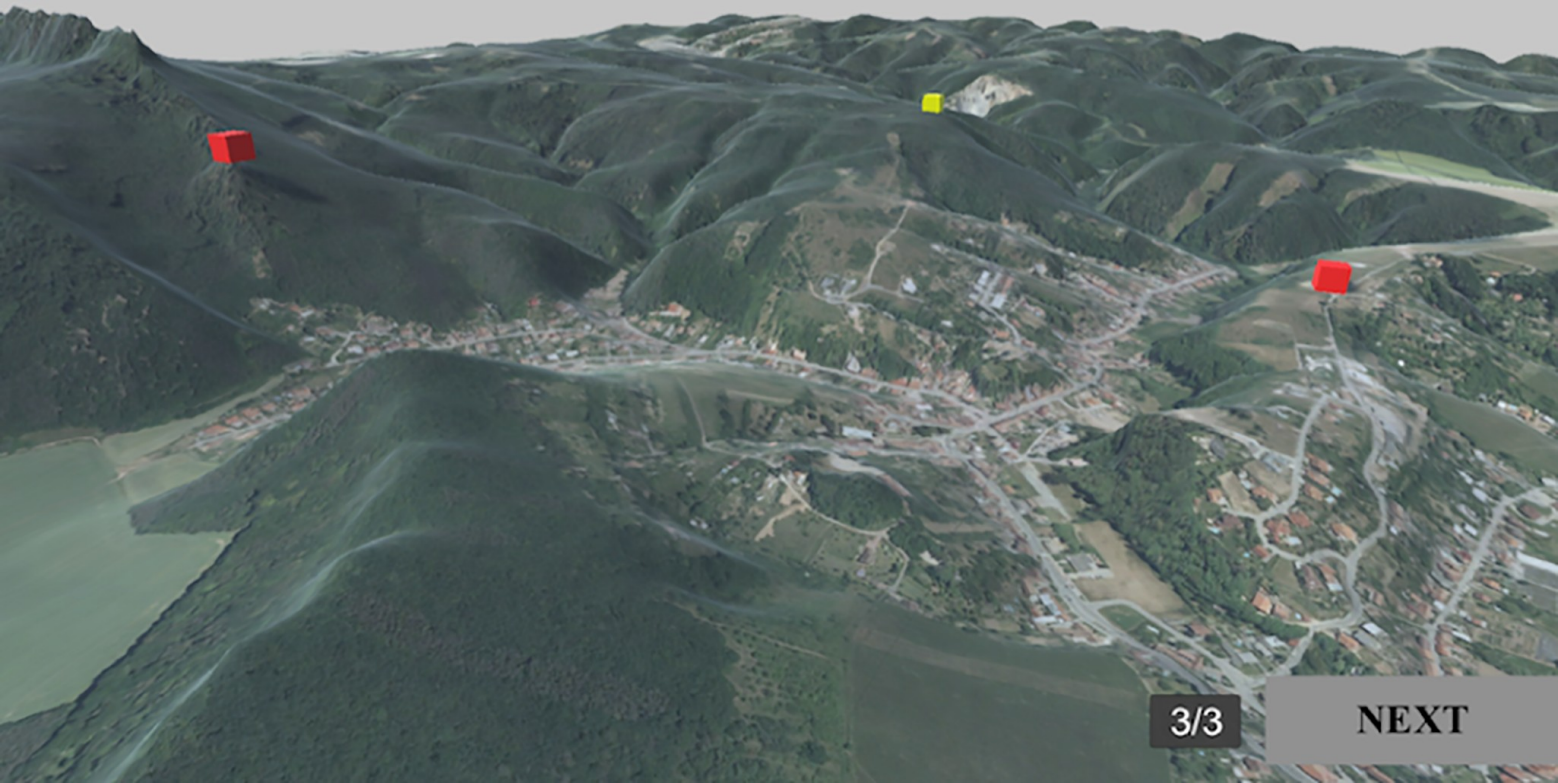
Example of Task 1 –Cubes in the terrain. **Yellow indicates that this object was selected by the participant.** Adapted from [61] under a CC BY license, with permission from [ČÚZK], original copyright [2018/2019].

Participants were asked to identify a cube placed at the highest elevation in the digital terrain model. Participants were shown a virtual scene with three cubes of the same red colour and asked to identify which one was located at the highest elevation. The cubes represented, for example, buildings. As point-symbols, buildings are considered the simplest spatial structure. Participants could identify the cubes with a mouse click. After clicking, the cube turned yellow, and participants could confirm their answer by clicking the “NEXT” button (Fig 3). Participants could also change their answers by clicking on one of the other cubes before pressing the “NEXT” button.

#### Comparing the positions of water bodies in the terrain

**Fig 4:**
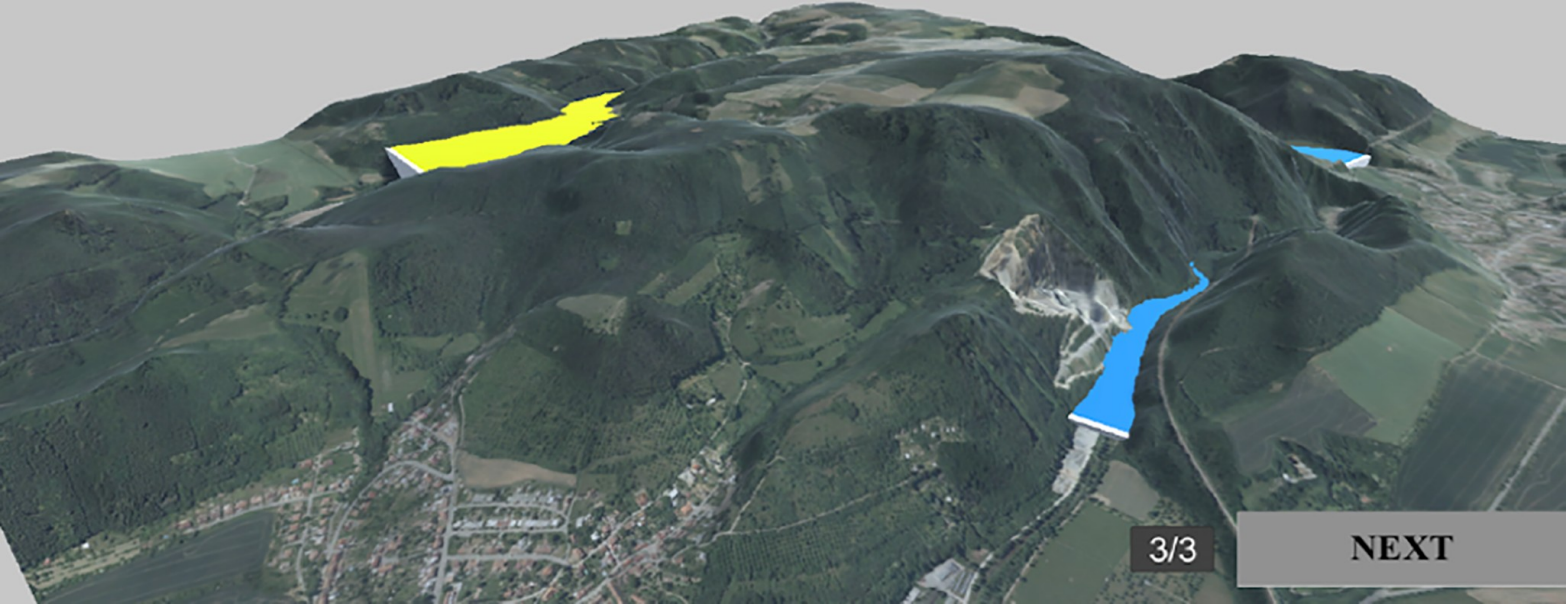
Example of Task 2 –Water bodies in the terrain. Yellow indicates that this object was selected by the participant. Adapted from [61] under a CC BY license, with permission from [ČÚZK], original copyright [2018/2019].

Participants were asked to identify the water body located at the highest elevation in the digital terrain model. Participants were shown a scene with three structures, illustrated as water bodies with the same blue colour, and asked to select which was positioned highest. Water bodies have a surface character, and in terms of their complexity, are considered stimuli between point symbols and areal symbols. As in the cube tasks, responses were made with a mouse click, turning the flat area yellow (Fig 4), and the task was finished by confirming the choice with the “NEXT” button.

#### Identifying terrain profiles

**Fig 5:**
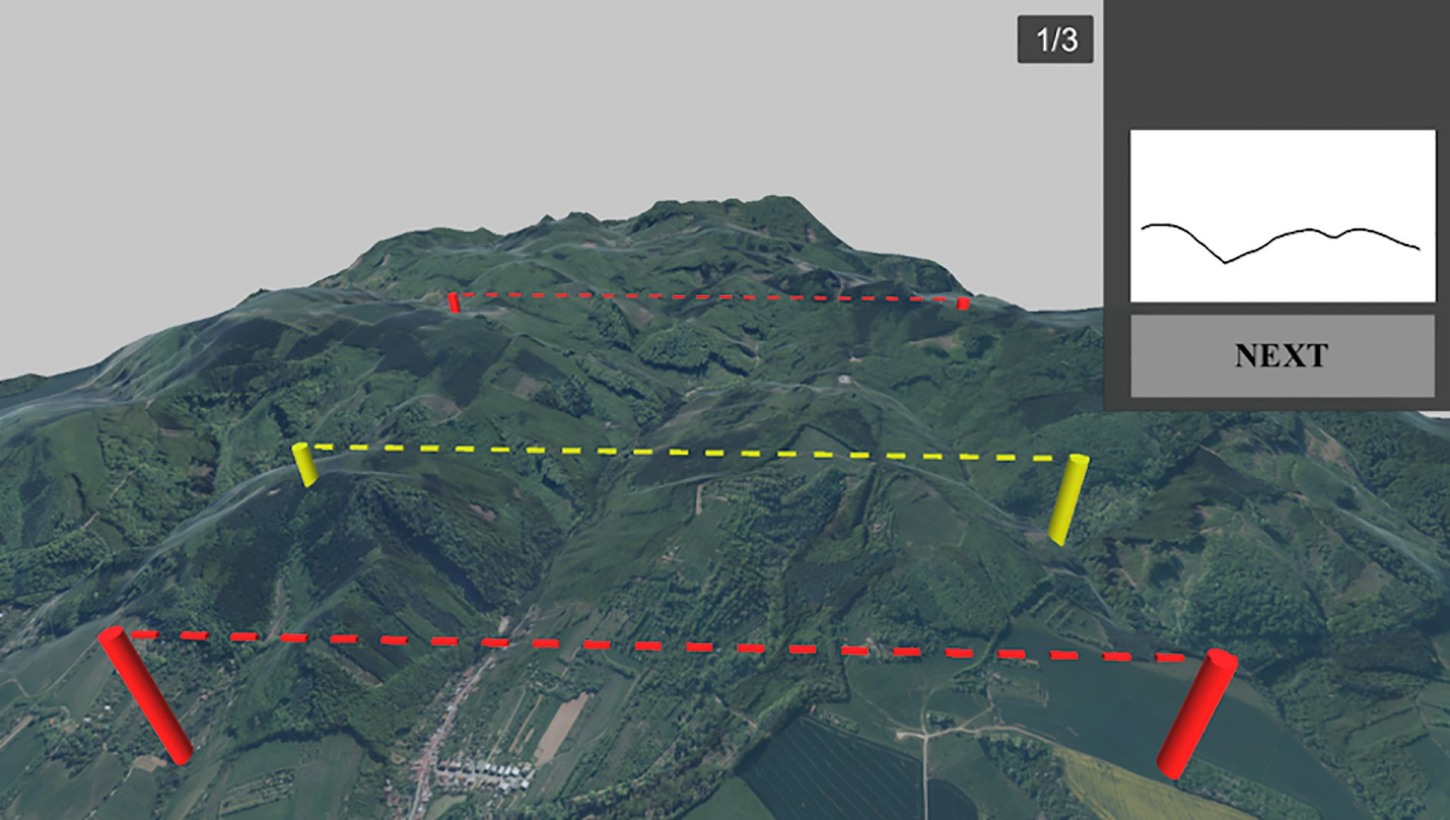
Example of Task 3 –Terrain profiles. Yellow indicates that the profile was selected by the participant. Adapted from [61] under a CC BY license, with permission from [ČÚZK], original copyright [2018/2019].

Participants were shown a scene with three structures located in the digital terrain model, each one representing a specific terrain profile between two points. A terrain profile presented as a 2D curve was displayed on the right side of the screen. Participants were asked to identify which of the three terrain profiles conformed to the shape of the curve depicted on the right. Since the elevation curve (complicated 3D structure) was not depicted, i.e. needed to be mentally computed, profile tasks are considered the most difficult. Again, as in the previously described tasks, the choice was made with a mouse click, turning the selected profile yellow (Fig 5), and the final answer was confirmed with the “NEXT” button.

### Stimuli

An original testing application based on the Unity^®^ game engine was developed for the experiment. This application renders large 3D terrain models in real-time and automatically collects data for further analysis. The 3D models of terrain were generated from a fourth-generation Digital Terrain Model of the Czech Republic (DTM 4G), which was originally created from airborne laser scanning and processed at a ground resolution of 5 × 5 m. DTM 4G is distributed by the ČÚZK (Czech Office for Surveying, Mapping and Cadastre). The terrains were selected so that the relief zones were similar in all territories. The study proceeded from a geographical regionalization of the Czech Republic [62], and all terrains depicted were highlands (relative height variation of 200 to 300 m). Terrains were covered with corresponding orthophotography data [61]. The 3D models were vertically scaled with a fixed factor of 3.0. Non-interactive (static) expositions were designed with respect to the position of the virtual camera from which the scene was viewed—screenshots of the terrains were created from two specific angles (45 degrees and 75 degrees), and the virtual camera was maintained at the same height in all static scenes. These static scenes were further used in testing, and the interactive scene was rendered in the real time.

### Analysis

As the dependent variables, the researchers analysed response times (RTs), correct elevation estimation (scores) and the number of mouse-clicks (clicks under interactive conditions). Correct answers scored 1, and incorrect answers scored 0. The RTs indicated asymmetric distribution, therefore linear mixed effect (LMER) modelling [63] was applied for analysis (due to the leftward inclination, a box-cox transformation was applied to the response time data). Score data were analysed with the use of generalized linear mixed effect models (GLMER), with logit as a link function and raw correctness as the dependent variable (1 = correct, 0 = incorrect). For the response times and scores, the visualization type (real 3D, pseudo 3D), interactivity type (interactive, static) and task type (cubes, water bodies and terrain profiles) and their interactions were considered fixed factors. The experiment included individual participants (according to their assigned identification numbers) as random intercepts. The models were executed using R software [64], R packages lme4 [65] and lmerTest [66]. The *p-*values for fixed effects were obtained using Satterthwaite approximation for degrees of freedom. All reported confidence intervals were obtained via bootstrapping (100000 iterations for LMER and 1000 iterations for GLMER) [63]. The mouse clicks were checked with the use of Spearman correlations to search for possible correspondence between RT and correctness.

## Results

### Response time

Overall, the linear mixed effects model for RTs revealed significant main effects of visualization type (*t* = 4.64, *p* < 0.001) and interaction type (*t* = -10.849, *p* < 0.001). Specifically, the real 3D environment increased the response time by 0.23 s ± 0.05 (95% CI [0.13309, 0.32925), and the option to interact prolonged response time by 0.54 s (se = 0.05s), 95% CI [-0.63256, -0.43963]. The main effect of the task type was also significant (*t* = 9.56, *p* < 0.001). Terrain profiles took 0.88 s ± 0.09 longer to solve than cubes (95% CI [0.69630, 1.05498]) and water bodies took 0.31 s ± 0.09 longer to solve than cubes (95% CI [0.13034, 0.48149]). The interaction effect of task and visualization factors was significant (*t* = -2.49, *p* = 0.0130) in water bodies compared to cubes. Real 3D visualization significantly reduced the response time in water bodies by 0.18 s ± 0.07 (95% CI [-0.31702, -0.03610]) and terrain profiles compared to cubes (*t* = -2.34, *p* = 0.0195). The 3D visualization significantly reduced the response time in terrain profiles by -0.17 s ± 0.07 (CI [-0.30700, -0.02639]). The interaction factor prolonged the response time in terrain profiles compared to cubes *(t* = 2.50, *p* = 0.0126) by 0.18 s ± 0.07 ([0.03815, 0.31652]). No other fixed effects were significant regarding response time (*p* > 0.5).

**Fig 6:**
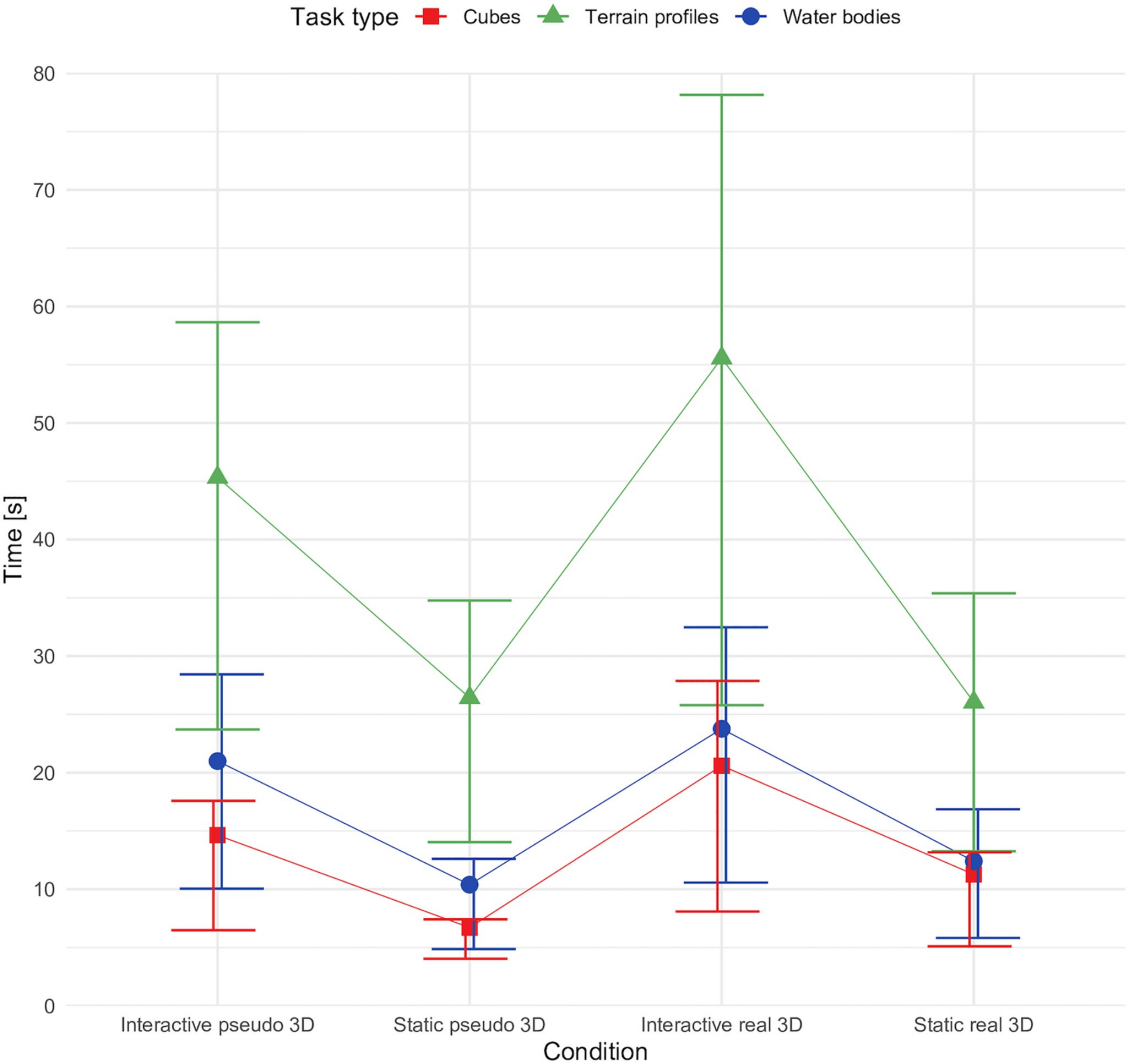
Participants’ response times according to conditions.

Fig 6 highlights that the response times exactly matched the expected trends of interactivity and real 3D visualization prolonging the task solving process. As the most spatially complex tasks, the terrain profiles took longest to solve, while cubes were solved the quickest. The largest difference in speed was observed between cubes solved in static pseudo 3D conditions and terrain profiles in interactive real 3D conditions.

### Correctness

Generalized linear mixed effects modelling (the Hosmer–Lemeshow Test suggested adequate goodness of fit: χ^2^ = 3.3563, *df* = 8, *p* = 0.91) on correctness revealed a significant main effect for interaction type (*β* = 1.842, *z* = 5.38, *p* < 0.001, 95% CI [1.30245, 2.40780]), suggesting that the static tasks were overall solved with greater accuracy. Water bodies were solved with greater accuracy than cubes (*β* = 0.564, *z* = 2.23, *p* = 0.0258, 95% CI [0.08624, 1.05926]). The interaction effects were significant for visualization type and interaction type (*β* = -2.232, *z* = -5.30, *p* < 0.001, 95% CI [-2.88130, -1.49627]). Interactive pseudo 3D conditions tended to encourage greater accuracy than static real 3D conditions. The effect of interaction was also observed in interaction type and task type in the case of terrain profiles (*β* = -1.376, *z* = -3.16, *p* = 0.0016). The terrain profiles were solved with less accuracy than cubes (95% CI [-2.09665, -0.65452]), and water bodies were solved with less accuracy than cubes (*β* = -2.318, *z* = -5.40, *p* < 0.001, 95% CI [-3.01544, -1.70323]). A triple interaction effect was observed in terrain profile tasks (*β* = 2.624, *z* = 4.57, *p* < 0.001), 95% CI [1.73593, 3.49377]), with a trend demonstrating that the terrain profiles were solved with greater accuracy in static real 3D conditions than cubes. The same trend was observed for water bodies (*β* = 1.670, *z* = 0.557, *p* < 0.001), where water bodies were solved with greater accuracy than cubes (95% CI [0.76593, 2.52489]) in the static real 3D condition.

**Fig 7:**
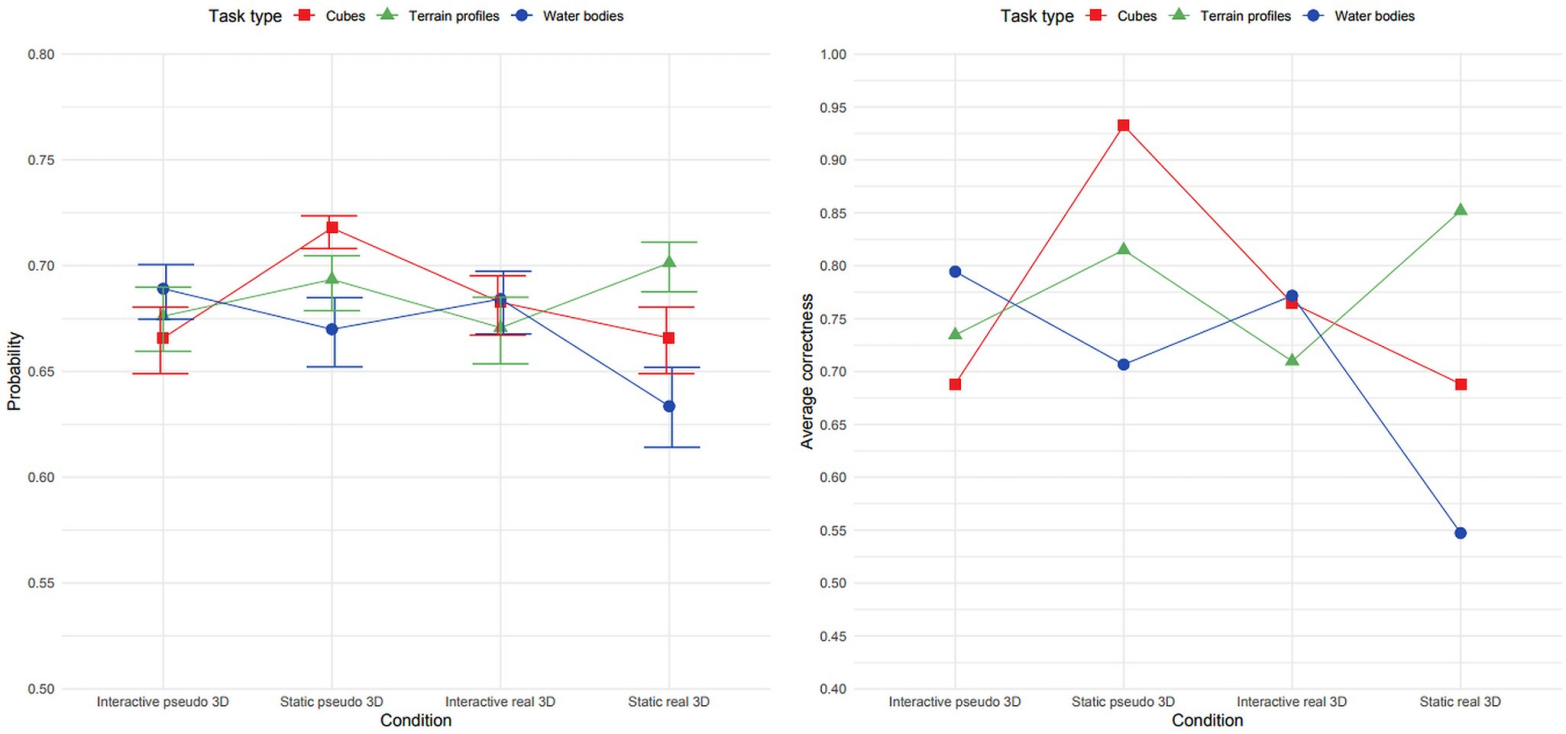
Calculated probabilities of accurate scoring according to conditions (left); participants’ average correctness scores (right).

Fig 7 (left) depicts the probabilities of accurate responses in different types of tasks with regards to the specific 3D and interactive condition, suggesting that no specific pattern between performance, tasks and conditions existed for correctness. The correctness scores in Fig 7 (right) illustrate that the study’s expectations on participant performance were not met and barely corresponded to the trends indicated by the time responses. The visual trend in both static conditions suggested a greater dispersion between individual types of tasks (cubes, water bodies, terrain profiles). Close values in the interactive conditions demonstrated interactivity as a feature that eliminated extreme values in participant performance.

### Mouse clicks

**Fig 8:**
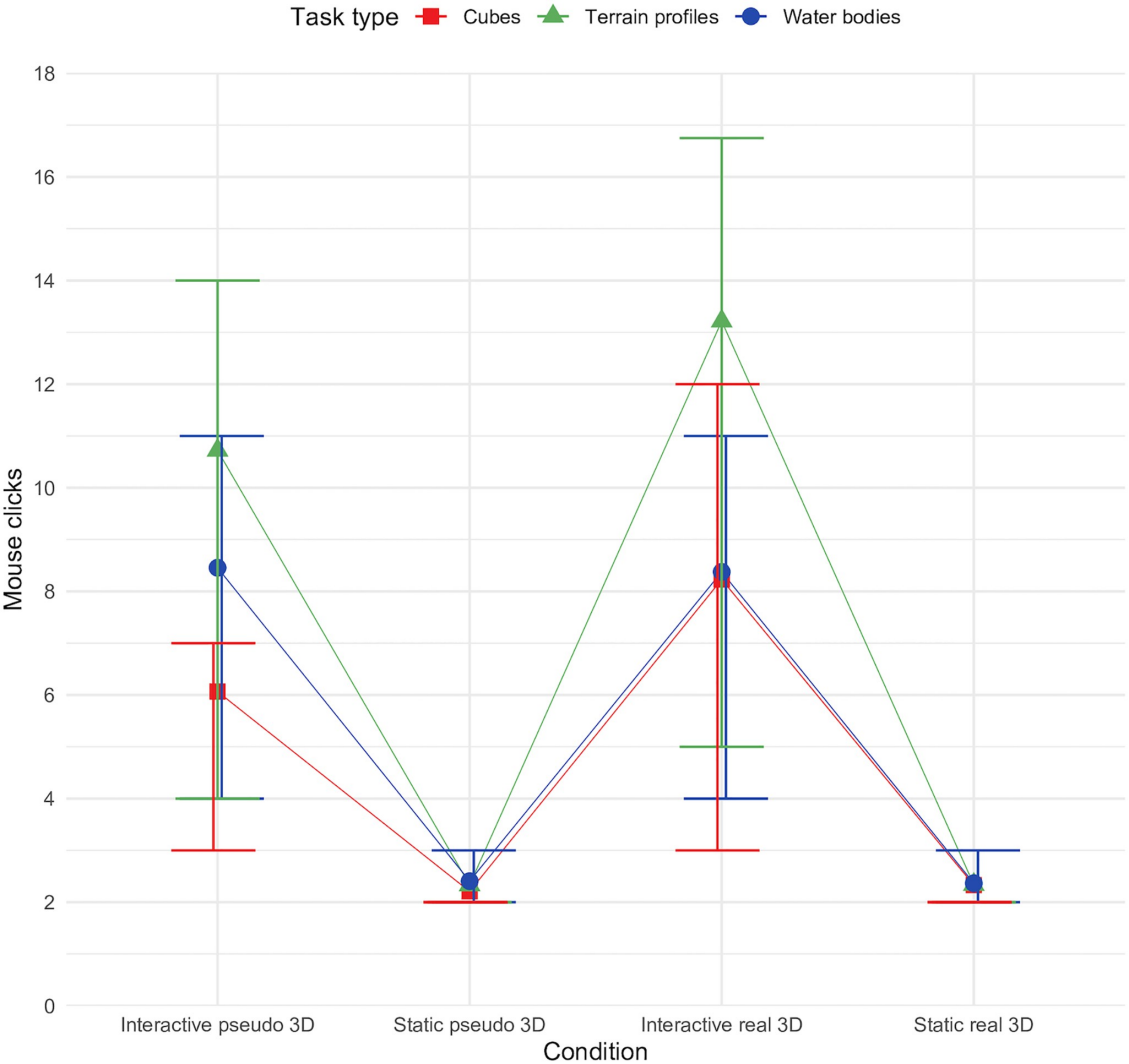
Number of mouse clicks according to the conditions.

Fig 8 charts the frequency of mouse clicks in the interactive tasks. The graph also contains data from static tasks. The number of mouse clicks participants performed while solving tasks in a specific condition corresponded to the response times, especially with respect to specific task type. A significant close positive correlation between RT and the number of mouse clicks was observed (*Spearman’s rho* = 0.827, *p* < 0.001). No correlation or trend was observed between correctness and the number of mouse clicks (*Spearman’s rho* = -0.056, *p* = 0.079). Generally, visual inspection suggested that more clicks were done in real 3D tasks, and the number of mouse clicks had an increasing trend in the terrain profiles tasks (Fig 8). The frequency of mouse clicks was lowest in the cube tasks.

## Discussion

The study examined the effectivity of stereoscopic real 3D visualizations and pseudo 3D visualizations under static and interactive conditions. Several effects were observed that contributed to the participants’ correctness and response times in solving the tasks. The number of mouse clicks to answer questions was also inspected.

### Response time

The response time patterns generally corresponded to the expectations that interactive and real 3D tasks would take longer to solve. This trend was consistent across all tasks. Participants who used the real 3D visualization generally took longer to solve the tasks (*t* = 4.63, *p* < 0.001). This effect was expected based on previous studies [23, 28, 35] and illustrated the possible increase in information load using 3D glasses and reading 3D maps. Similarly, an increase in response time was observed in the case of interactivity, participants spending more time solving interactive tasks (*t* = -10.849, *p* < 0.001). This effect was also expected based on previous studies [36, 49], which suggested interactive tasks were more engaging and time consuming. Both findings met the study’s expectations on the effect of 3D visualization in geovisualizations. Regarding the speed of responses, a clear trend was identified with respect to the specific task types. The terrain profile tasks took participants significantly more time than other tasks, indicating that spatially complex tasks, such as assessing elevation relationships in the terrain, require more time to process than spatially simple scenes (e.g. point structures in the terrain). The effect of interaction on task type and visualization type factors was significant (t = -2.49, *p* = 0.0130) in water bodies compared to cubes, where real 3D visualization significantly decreased the response time in water bodies. In the case of terrain profile tasks compared to cubes, the 3D visualization significantly reduced the response time in terrain profiles (t = -2.34, p = 0. 0195). This may indicate that 3D visualizations can potentially increase efficiency in spatially challenging tasks, i.e. when a task is spatially more complex, and that real 3D visualization tools may assist in solving tasks more quickly.

### Correctness

The empirical results show that the interactive tasks were generally solved with significantly less accuracy than the static tasks (*β* = 1.842, *z* = 5.38, *p* < 0.001), which did not confirm the hypothesis of a generally higher effectivity in an interactive setting (e.g. [49]). Since a positive effect was identified from interactivity in pseudo 3D conditions (*β* = -2.231, *z* = -5.29, *p* < 0.001), interactivity seemingly has the potential to promote spatial assessment of virtual terrains in pseudo 3D settings. From this point of view, interactivity can be seen as complementing the missing binocular depth cues in pseudo 3D visualizations. Regarding the interactivity factor, the cubes were solved with greater accuracy in interactive conditions than in terrain profiles (*β* = -1.376, *z* = -3.15, p = 0. 0016), and then water bodies (*β* = -2.318, *z* = -5.40, *p* < 0.001), which illustrates the increasing effectivity of interactivity in the cube tasks (spatially simple structures). A triple interaction effect was observed in terrain profile tasks (*β* = 2.624, *z* = 4.57, *p* < 0.001), with a trend demonstrating that the terrain profiles were solved with greater accuracy in real 3D static condition than cubes. The same trend was observed in the case of water bodies (*β* = 1.670, *z* = 0.557, *p* < 0.001). Water bodies were solved with greater accuracy in static real 3D conditions than cubes, illustrating the positive effect of real 3D visualization in tasks based on elevation profiles (i.e. spatially complex structures). In the static conditions, greater differences (dispersion) were observed in the participants’ performance in all three types of tasks, however the participants’ performance in the interactive conditions was similar, which indicates that interactivity may have the potential to eliminate extreme values and also emphasize the specific factors of task types, i.e. that the concrete settings may encourage specific task solving. This finding corresponds to previous research [45], which concluded that the type of task contributed a more important role in static conditions than interactive settings, also highlighting the need for task-focused research.

### Mouse clicks

The frequency of mouse clicks in the interactive tasks, not surprisingly, corresponded to the response times. A significant close positive correlation was observed between the RT and the number of mouse clicks (*p* < 0.001). This finding was not surprising considering that interaction itself takes time and corresponded to previous findings [49]. No significant correlation or trend was observed between correctness and the number of mouse clicks (*Spearman’s rho* = -0.056, *p* = 0.079). Generally, the visual inspection suggested that real 3D conditions promoted mouse-clicking, i.e. the tendency to interact with the scene. The number of mouse clicks demonstrated an increasing trend in the terrain profiles tasks (Fig 8), which can be classified as spatially more complex, demanding greater interaction.

The empirical findings appear counterintuitive in several ways, especially regarding the correctness scores, as no clear pattern indicated the advantage of a specific factor in the participants’ correctness performance. Regarding the previous research, it can be suggested that this indicates a lack of participants’ attention or the motivation to find easy-looking tasks. To encourage confidence in their responses, participants were not motivated to collect more information about the scene, which might have also led to errors. A similar effect was discussed in [36, 52]. This could be considered a “metacognitive mistake”, i.e. anticipating a task as easy may have decreased the participants’ mental efforts, thereby reducing their full use of the potentially available information. No clear pattern indicated the dependence between response time and correctness. The general trend in the correctness scores did not correspond to the solving speed. This speed-accuracy trade-off may have reframed what participants thought was the purpose of the tasks and encouraged them to guess the answers. The participants may have focused more on speed than accuracy and responded with the first available option. The pattern of response time scores may simply have emulated the primary expectations for interface properties in geovisualizations, i.e. that interaction takes more time and real 3D is visually more difficult, therefore the RT performance corresponded to the ease of use.

## Conclusion

In the present study, real 3D visualization was compared to pseudo 3D visualization in a digital terrain model experiment. The efficiency and effectiveness of stereoscopic real 3D visualization was examined in both static and interactive conditions, using three types of task as experimental stimuli representing different geo-related phenomena. The results suggested that real 3D visualization (*t* = 4.64, *p* < 0.001) and the interactivity option (*t* = -10.849, *p* < 0.001) increased response time. Counterintuitively, our data demonstrated that the static condition increased response correctness (*β* = 1.842, *z* = 5.38, *p* < 0.001). With respect to the more detailed analyses of factor interactions in the presented models, it can be summarized that interactivity has the potential to increase performance when a pseudo 3D visualization is applied (potentially complementing real 3D binocular depth cues) and that real 3D visualization may increase performance in tasks dealing with more complex terrain shapes, i.e. structures such as terrain profiles, which require deeper mental computation. Generally, it can be concluded that 3D visualizations using geospatial data is a broad issue and influenced by various factors, the most important factors being the type of visualization, interactivity, specific applications, task type, and also the user group aspects. As concluded in previous studies [30, 36, 41, 49], the benefits and limitations of stereoscopic 3D visualizations are still not completely clear in the various applications of geo-sciences, especially regarding the interactivity factor. Future research should explore interactive and advanced geospatial tasks in terms of the discussed theory as well as the present study’s findings that the option to interact and the specific nature of the task may strongly affect the cognitive processing of the presented stimuli.

## References

[1] Hirmas DR., Slocum T, Halfen AF, White T, Zautner E, Atchley P, et al Effects of Seating Location and Stereoscopic Display on Learning Outcomes in an Introductory Physical Geography Class. Journal of Geoscience Education 2014; 62(1): 126–137. 10.5408/12-362.1

[2] Philips A, Walz A, Bergner A, Graeff T, Heistermann M, Kienzler S, et al Immersive 3D Geovisualization in Higher Education. Journal of Geography in Higher Education 2015; 39(3), 437–449. 10.1080/03098265.2015.1066314

[3] Stojšić I, Ivkov Džigurski A, Maričić O, Ivanović Bibić L, Đukičin Vučković S. Possible Application of Virtual Reality in Geography Teaching. Journal of Subject Didactics 2017; 1(2): 83–96. 10.5281/zenodo.438169

[4] Carbonell-Carrera C, Saorín J. Geospatial Google Street View with Virtual Reality: A Motivational Approach for Spatial Training Education. ISPRS International Journal of Geo-Information 2017, 6(9): 1–10. 10.3390/ijgi6090261

[5] Šašinka Č, Stachoň Z, Sedlák M, Chmelík J, Herman L, Kubíček P, et al Collaborative Immersive Virtual Environments for Education in Geography. ISPRS International Journal of Geo-Information 2019; 8(1): 1–25. 10.3390/ijgi8010003

[6] Abulrub A-HG, Budabuss K, Mayer P, Williams MA. The 3D Immersive Virtual Reality Technology Use for Spatial Planning and Public Acceptance. Procedia—Social and Behavioral Sciences 2013; 75(2013): 328–337. 10.1016/j.sbspro.2013.04.037

[7] Jamei E, Mortimer M, Seyedmahmoudian M, Horan B, Stojcevski A. Investigating the Role of Virtual Reality in Planning for Sustainable Smart Cities. Sustainability 2017; 9(11): 1–16; 10.3390/su9112006

[8] Afrooz AE, Lowe R, Leao SZ, Pettit C. 3D and Virtual Reality for Supporting Redevelopment Assessment In: Reed R, Pettit C, editors. Real Estate and GIS, 2018. London, UK: Routledge pp. 162–185. 2018 10.1201/9781315642789-9

[9] Tang C-H, Wu W-T, Lin C-Y. Using Virtual Reality to Determine How Emergency Signs Facilitate Way-finding. Applied Ergonomy 2009; 40(4): 722–730. 10.1016/j.apergo.2008.06.009 18708182

[10] Smith S, Ericson E. Using Immersive Game-based Virtual Reality to Teach Fire-safety Skills to Children. Virtual Reality 2009; 13: 87–99. 10.1007/s10055-009-0113-6

[11] Meng F, Zhang W. Way-finding during a Fire Emergency: an Experimental Study in a Virtual Environment. Ergonomics 2014; 57(6): 816–827. 10.1080/00140139.2014.904006 24697193

[12] Preppernau CA, Jenny B. Three-dimensional versus Conventional Volcanic Hazard Maps. Natural Hazards 2015; 78(2): 1329.1347 10.1007/s11069-015-1773-z

[13] Charvát K, Řezník T, Lukas V, Charvát K jr, Jedlička K, Palma R, et al Advanced Visualisation of Big Data for Agriculture as Part of DataBio Development In: IGARSS 2018–2018 IEEE International Geoscience and Remote Sensing Symposium (pp. 415–418). Valencia, Spain: IEEE 2018 10.1109/IGARSS.2018.8517556

[14] Chmielewski S, Tompalski P. Estimating Outdoor Advertising Media Visibility with Voxel-based Approach. Applied Geography, 87(2017): 1–13. 10.1016/j.apgeog.2017.07.007

[15] Savova D, Bandrova T. 3D Mapping of Mountain Territories—Virtual Visualization by 3D Symbol System In: Bandrova T, Koneč M, editors. Proceedings of the 5th International Conference on Cartography and GIS (pp. 388–396). Riviera, Bulgaria: Bulgarian Cartographic Association 2014.

[16] McKenzie G, Klippel A. The Interaction of Landmarks and Map Alignment in You-Are-Here Maps. Cartographic Journal 2016; 53(1): 43–54. 10.1179/1743277414Y.0000000101

[17] Liao H, Dong W. An Exploratory Study Investigating Gender Effects on Using 3D Maps for Spatial Orientation in Wayfinding. ISPRS International Journal of Geo-Information 2017, 6(3): 1–19. 10.3390/ijgi6030060

[18] Zhou Y, Dao THD, Thill J-C, Delmelle E. Enhanced 3D Visualization Techniques in Support of Indoor Location Planning. Computers, Environment and Urban Systems 2016; 50(2015): 15–29. 10.1016/j.compenvurbsys.2014.10.003

[19] Snopková D, Švedová H, Kubíček P, Stachoň Z. Navigation in Indoor Environments: Does the Type of Visual Learning Stimulus Matter?. ISPRS International Journal of Geo-Information 2019; 8(6): 1–26. 10.3390/ijgi8060251

[20] Bleisch S. 3D Geovisualization—Definition and Structures for the Assessment of Usefulness. ISPRS Annals of Photogrammetry, Remote Sensing and Spatial Information Sciences 2012 I-2 129–134. 10.5194/isprsannals-I-2-129-2012

[21] MacEachren AM, Kraak MJ. Research Challenges in Geovisualization Cartography and Geographic Information Science 2001, 28(1): 3–12. 10.1559/152304001782173970

[22] Buchroithner MF, Knust C. True-3D in Cartography. Current Hard and Softcopy Developments In: Moore A, Drecki I, editors. Geospatial Visualisation, 2013 Heidelberg, Germany: Springer pp. 41.65 10.1007/978-3-642-12272-9

[23] Seipel S. Evaluating 2D and 3D Geovisualisations for Basic Spatial Assessment. Behaviour & Information Technology 2013; 32(8): 845–858. 10.1080/0144929X.2012.661555

[24] Aflaki P, Hannuksela M, Sarbolandi H, Gabbouj M. Simultaneous 2D and 3D Perception for Stereoscopic Displays based on Polarized or Active Shutter Glasses. Journal of Visual Communication and Image Representation 2014; 25(4): 622–631. 10.1016/j.jvcir.2013.03.014

[25] Choi H. Current Status of Stereoscopic 3D LCD TV Technologies. 3D Research 2011; 2(2): 1–4. 10.1007/3DRes.02(2011)4

[26] Qian N. Binocular Disparity and the Perception of Depth. Neuron 1997; 18(3): 359–368. 10.1016/s0896-6273(00)81238-6 9115731

[27] Landy M, Maloney L, Johnston E, Young M. Measurement and Modeling of Depth Cue Combination: in Defense of Weak Fusion. Vision Research 1995; 35(3): 389–412. 10.1016/0042-6989(94)00176-m 7892735

[28] Livatino S, De Paolis LT, D’Agostino M, Zocco A, Agrimi A, De Santis A, et al Stereoscopic Visualization and 3-D Technologies in Medical Endoscopic Teleoperation. IEEE Transactions on Industrial Electronics 2015; 62(1): 525–535. 10.1109/TIE.2014.2334675

[29] Weber A, Jenny B, Wanner M, Cron J, Marty P, Hurni L. Cartography Meets Gaming: Navigating Globes, Block Diagrams and 2D Maps with Gamepads and Joysticks. Cartographic Journal 2010; 47(1): 92–100. 10.1179/000870409X12472347560588

[30] Torres J, Ten M, Zarzoso J, Salom L, Gaitán R, Lluch J. Comparative Study of Stereoscopic Techniques Applied to a Virtual Globe. Cartographic Journal 2013; 50(4): 369–375. 10.1179/1743277413Y.0000000034

[31] Dušek R, Miřijovský J. Vizualizace prostorových dat: chaos v dimenzích [Visualization of Geospatial Data: Chaos in the Dimensions]. Geografie 2009, 114(3): 169–178.

[32] IEEE 610:1990. IEEE Standard Computer Dictionary: A Compilation of IEEE Standard Computer Glossaries. Piscataway, NJ, USA: IEEE 1990.

[33] Rubin J, Chisnell D, Spool J. Handbook of Usability Testing: How to Plan, Design, and Conduct Effective Tests. 2nd ed Hoboken, NJ, USA: Wiley 2008.

[34] Van Beurden MHPH, Kuijsters A, Ijsselsteijn WA. Performance of a Path Tracing Task Using Stereoscopic and Motion based Depth Cues In: Second International Workshop on Quality of Multimedia Experience (QoMEX) (pp. 176–18). Trondheim, Norway: IEEE 2010 10.1109/QOMEX.2010.5516268

[35] Rydmark M, Kling-Petersen T, Pascher R, Philip F. 3D Visualization and Stereographic Techniques for Medical Research and Education. Studies in Health Technology and Informatics 2001 81: 434–439. 10.3233/978-1-60750-925-7-434 11317785

[36] Juřík V, Herman L, Šašinka Č, Stachoň Z, Chmelík J. When the Display Matters: A Multifaceted Perspective on 3D Geovisualizations. Open Geosciences 2017, 9(1): 89–100. 10.1515/geo-2017-0007

[37] Fuhrmann S, Komogortsev O, Tamir D. Investigating Hologram-Based Route Planning. Transactions in GIS 2009, 13(s1): 177–196. 10.1111/j.1467-9671.2009.01158.x

[38] Kjellin A, Pettersson LW, Seipel S, Lind M. Evaluating 2D and 3D Visualizations of Spatiotemporal Information. ACM Transactions on Applied Perception 2010; 7(3): 1–23. 10.1145/1773965.1773970

[39] Seipel S, Carvalho L. Solving Combined Geospatial Tasks Using 2D and 3D Bar Charts In: 16th International Conference on Information Visualisation (pp. 157–163). Montpellier, France: IEEE 2012 10.1109/IV.2012.36

[40] Edler D, Bestgen A, Kuchinke L. Dickmann F. Grids in Topographic Maps Reduce Distortions in the Recall of Learned Object Locations. PLoS ONE 2014, 9(5): 1–10. 10.1371/journal.pone.0098148 PMC4037198 24869486

[41] Zanola S, Fabrikant SI, Çöltekin A. The Effect of Realism on the Confidence in Spatial Data Quality in Stereoscopic 3D Displays In: Proceedings, 24th International Cartography Conference (ICC 2009). Santiago: ICA 2009 http://www.geo.uzh.ch/~sara/pubs/zanola_fabrikant_coeltekin_icc09.pdf

[42] Shepherd I. Travails in the Third Dimension: A Critical Evaluation of Three Dimensional Geographical Visualization In: Dodge M, McDerby M, Turner M, editors. Geographic Visualization: Concepts, Tools and Applications; 2008 Hoboken, NJ, USA: Wiley pp. 199–222.

[43] Roth RE. Cartographic Interaction Primitives: Framework and Synthesis. Cartographic Journal 2012; 49(4): 376–395. 10.1179/1743277412Y.0000000019

[44] Carbonell-Carrera C. Spatial-Thinking Knowledge Acquisition from Route-Based Learning and Survey Learning: Improvement of Spatial Orientation Skill with Geographic Information Science Sources. Journal of Surveying Engineering 2017, 143(1), 05016009 10.1061/(asce)su.1943-5428.0000200

[45] Herman L, Juřík V, Stachoň Z, Vrbík D, Russnák J, Řezník T. Evaluation of User Performance in Interactive and Static 3 D Maps. ISPRS International Journal of Geo-Information 2018; 7(11): 1–25. 10.3390/ijgi7110415

[46] Carbonell-Carrera C, Gunalp P, Saorin JL, Hess-Medler S. Think Spatially With Game Engine. ISPRS International Journal of Geo-Information 2020, 9(3), 159 10.3390/ijgi9030159

[47] Bleisch S, Dykes J, Nebiker S. Evaluating the Effectiveness of Representing Numeric Information through Abstract Graphics in 3D Desktop Virtual Environments. Cartographic Journal 2013; 45(3): 216–226. 10.1179/000870408X311404

[48] erbert G, Chen X. A Comparison of Usefulness of 2D and 3D Representations of Urban Planning. Cartography and Geographic Information Science 2014, 42(1): 22–32. 10.1080/15230406.2014.987694

[49] Kubíček P, Šašinka Č, Stachoň Z, Herman L, Juřík V, Urbánek T, et al Identification of Altitude Profiles in 3D Geovisualizations: The Role of Interaction and Spatial Abilities. International Journal of Digital Earth 2019; 12(2): 156–172. 10.1080/17538947.2017.1382581

[50] Bingham G, Lind M. Large Continuous Perspective Transformations are Necessary and Sufficient for Accurate Perception of Metric Shape. Perception & Psychophysics 2008; 70(3): 524–540. 10.3758/PP.70.3.524 18459264

[51] Rogers B, Graham M. Similarities between Motion Parallax and Stereopsis in Human Depth Perception. Vision research 1982; 22(2): 261–270. 10.1016/0042-6989(82)90126-2 7101762

[52] Keehner M, Hegarty M, Cohen C, Khooshabeh P, Montello D. Spatial Reasoning With External Visualizations: What Matters Is What You See, Not Whether You Interact. Cognitive Science: A Multidisciplinary Journal 2008; 32(7): 1099–1132. 10.1080/03640210801898177 21585445

[53] Liu B, Dong W, Meng L. Using Eye Tracking to Explore the Guidance and Constancy of Visual Variables in 3D Visualization. ISPRS International Journal of Geo-Information 2017, 6(9): 1–18. 10.3390/ijgi6090274

[54] Juřík V, Herman L, Šašinka Č, Stachoň Z, Chmelík J, Strnadová A, et al Behavior Analysis in Virtual Geovisualizations: Towards Ecological Validity In Bandrova T, Konečný M, editors. Proceedings of the 7th International Conference on Cartography and GIS (pp. 518–527). Sofia, Bulgaria: Bulgarian Cartographic Association, 2018.

[55] Ugwitz P, Juřík V, Herman L, Stachoň Z, Kubíček P, Šašinka Č. Spatial Analysis of Navigation in Virtual Geographic Environments. Applied Sciences 2019; 9(9): 1–22. 10.3390/app9091873

[56] Quesenbery W. What Does Usability Mean: Looking Beyond ‘Ease of Use’ In: Proceedings of the 48th Annual Conference, Society for Technical Communication (pp. 432–436). Chicago, USA: Society for Technical Communication 2001 https://www.wqusability.com/articles/more-than-ease-of-use.html

[57] Naepflin U, Menozzi M. Can Movement Parallax Compensate Lacking Stereopsis in Spatial Explorative Search Tasks?. Displays 2001; 22(2): 157–164. 10.1016/S0141-9382(01)00067-1

[58] Sollenberger RL, Milgram P. Effects of Stereoscopic and Rotational Displays in a Three-dimensional Path-tracing Task. Journal of the Human Factors and Ergonomics Society 35(3): 483–99. 10.1177/001872089303500306 8244411

[59] Ware C, Franck G. Evaluating Stereo and Motion Cues for Visualizing Information Nets in Three Dimensions. ACM Transactions on Graphics 1996; 15 (2): 121–140. 10.1145/234972.234975

[60] Faubert J. Motion Parallax, Stereoscopy, and the Perception of Depth: Practical and Theoretical Issues In: Proceedings of SPIE (pp. 168–191). Boston, USA 2001 10.1117/12.419794

[61] Český úřad zeměměřický a katastrální. Ortofoto; 2018/2019 © [cited 2020 Apr 5]. Ortofoto [Web Map Service]. https://geoportal.cuzk.cz/WMS_ORTOFOTO_PUB/WMService.aspx

[62] Kudrnovská O, Kousal J. Výšková členitost reliéfu ČSR, Mapa 1:500 000. Brno, ČSR: Geografický ústav ČSAV 1971.

[63] Kirby KN, Gerlanc D. BootES: An R Package for Bootstrap Confidence Intervals on Effect Sizes. Behavior Research Methods 2013; 45(4), 905–927. 10.3758/s13428-013-0330-5 23519455

[64] R Development Core Team. R. 2015.

[65] Bates DM, Mächler M, Bolker B, Walker S. Fitting Linear Mixed-Effects Models Using lme4. Journal of Statistical Software 2015; 67(1): 1–48. 10.18637/jss.v067.i01

[66] Kuznetsova A, Brockhoff PB, Christensen RHB. lmerTest Package: Tests in Linear Mixed Effects Models. Journal of Statistical Software 2017; 82(13). 10.18637/jss.v082.i13

